# Anxiety and determinants among patients with chronic diseases during pandemic of emerging infectious diseases: a moderated mediation analysis

**DOI:** 10.3389/fpubh.2025.1689980

**Published:** 2025-11-13

**Authors:** Yuxiao Wang, Sitian Li, Haiting Zheng, Yaodan Zhang, Jingru Zhang, Yuanyuan La, Jun Xie, Jiantao Li, Lu He

**Affiliations:** 1 Department of Health Economics, School of Management, Shanxi Medical University, Taiyuan, Shanxi, China; 2 Department of Social Medicine, School of Public Health, Shanxi Medical University, Taiyuan, Shanxi, China; 3 School of International and Public Affairs, Shanghai Jiao Tong University, Shanghai, China; 4 Center of Reverse Microbial Etiology, Shanxi Medical University, Taiyuan, Shanxi, China; 5 Key Laboratory of Coal Environmental Pathopoiesis and Control at Shanxi Medical University, Ministry of Education, Taiyuan, Shanxi, China

**Keywords:** chronic diseases, anxiety, moderated mediation model, public health crises, mental health outcomes

## Abstract

**Background:**

This study aims to investigate the anxiety levels and associated influencing factors among patients with chronic diseases during the pandemic of emerging and sudden infectious diseases. The findings are intended to provide a theoretical foundation for developing targeted psychological intervention strategies, thereby enhancing the mental health support for chronic disease patients in times of public health crises.

**Methods:**

This study was conducted in Shanxi Province between December 2022 and January 2023, utilizing a cross-sectional research design with a total sample size of 40,302 participants. Chi-square tests were employed to assess differences across groups in sociodemographic characteristics, prevalence of chronic diseases, vaccination status, self-perceived symptoms, availability of essential supplies, and anxiety levels. Additionally, a moderated mediation model was applied to examine the potential pathways through which these variables influence anxiety. All statistical analyses were carried out using R software.

**Results:**

Increasing the number of emerging and sudden infectious diseases vaccination doses negatively predicted anxiety (β = −0.0249, 95%CI = [−0.0393, −0.0106]), a relationship partially mediated by self-perceived symptoms (effect = 0.0102, SE = 0.002, 95%CI = [0.0062, 0.0142]). Personal material reserves and residential GDP significantly moderated anxiety (β = −0.035, 95%CI = [0.0426, −0.0273]; β = 0.033, 95%CI = [0.0127, 0.0533]). The effect of self-perceived symptoms on anxiety was greater in areas with lower GDP and among patients with chronic diseases with poorer material reserves.

**Conclusion:**

This study highlights the critical role of vaccination in reducing anxiety during public health emergencies, particularly among chronic disease patients, and identifies key factors such as self-perceived symptoms, material reserves, and local GDP. Strengthening vaccination coverage and securing essential supplies for high-risk groups are vital to safeguarding their mental health in such crises. These findings offer both theoretical and practical insights for addressing post-outbreak anxiety.

## 1 Introduction

Anxiety disorders are the sixth leading cause of disability globally (1), with the emerging and sudden infectious diseases pandemic having caused unprecedented stress owing to uncertainty and security concerns (2). People with non-communicable chronic diseases are at higher risk of developing anxiety (3).chronic diseases refers to conditions that are long-lasting (≥ 1 year), require ongoing medical care, and limit the activities of daily living (4). Patients with chronic diseases face physical and psychological difficulties owing to the persistent nature of associated symptoms. Chronic diseases, thus, have a significant impact on patients’ health-related quality of life and are associated with decreased functioning, increased risk of death, and increased costs of personal medical care (5, 6). These patients often exhibit psychological symptoms, such as anxiety and depression, which can have a further detrimental effect on their condition (7). Previous studies have shown that anxiety is associated with poor physical health outcomes and quality of life in patients with chronic diseases (8, 9), and care disruption as well as other challenges associated with the emerging and sudden infectious diseases pandemic may have similar effects (10).

More frequent vaccination doses not only contributes to enhancing herd immunity but also helps reduce transmission risk and mitigate community anxiety, especially in population clusters with underlying diseases (11). However, news about new variants, the need for booster shots, and evolving vaccine recommendations may also cause anxiety for certain individuals (12). In this study, we sought to further elucidate the psychological mechanisms underlying the association between emerging and sudden infectious diseases vaccination doses and anxiety; thus, Hypothesis 1 (H1) is proposed.

*H1*: Higher vaccination doses during periods of large-scale outbreaks of emerging and sudden infectious diseases infections predict lower levels of anxiety.

The severity of self-perceived symptoms can influence anxiety levels (13). Individuals experiencing more severe symptoms may worry more about potential adverse effects or health-related concerns. Self-perceived symptom severity acts as a mediator between vaccination and emerging and sudden infectious diseases -related anxiety. This influences the extent to which vaccination doses affect anxiety, with individuals experiencing milder symptoms likely to have lower anxiety levels by comparison. The mediating role of self-perceived symptom severity highlights the importance of personal experiences and perceptions following vaccination. Therefore, understanding the role of self-perceived symptoms in addressing and managing vaccine-related anxiety is crucial. Based on this assertion, Hypothesis 2 (H2) is proposed.

*H2*: Self-perceived symptoms mediate the association between the number of emerging and sudden infectious diseases vaccination doses and anxiety.

Previous research has shown that behaviors such as stockpiling masks, medication, and antiseptic solutions, as well as seeking professional medical care have occurred more frequently following the introduction of China’s new reduced emerging and sudden infectious diseases policy (14). Individuals with sufficient material reserves may feel more confident because they know that they can readily protect themselves in the event of another emerging and sudden infectious diseases outbreak, reducing their associated anxiety levels (15). This may also reduce anxiety resulting from perceived symptom severity, which informs Hypothesis 3 (H3).

*H3*: Material reserves play a moderating role between vaccination status and anxiety. Specifically, the association between self-perceived symptoms and anxiety is stronger in individuals with lower rather than higher material reserves.

Higher gross domestic product (GDP) areas tend to have a stronger economic base, which may grant easier access to the medical resources and support needed to respond to an outbreak (16). Thus, living in a relatively affluent, well-resourced area may help alleviate people’s concerns. Therefore, Hypothesis 4 (H4) is proposed.

*H4*: Residential GDP plays a moderating role between vaccination status and anxiety. Specifically, the association between self-perceived symptoms and anxiety is stronger in individuals with lower residential GDP than in those with higher residential GDP.

**Figure 1:**
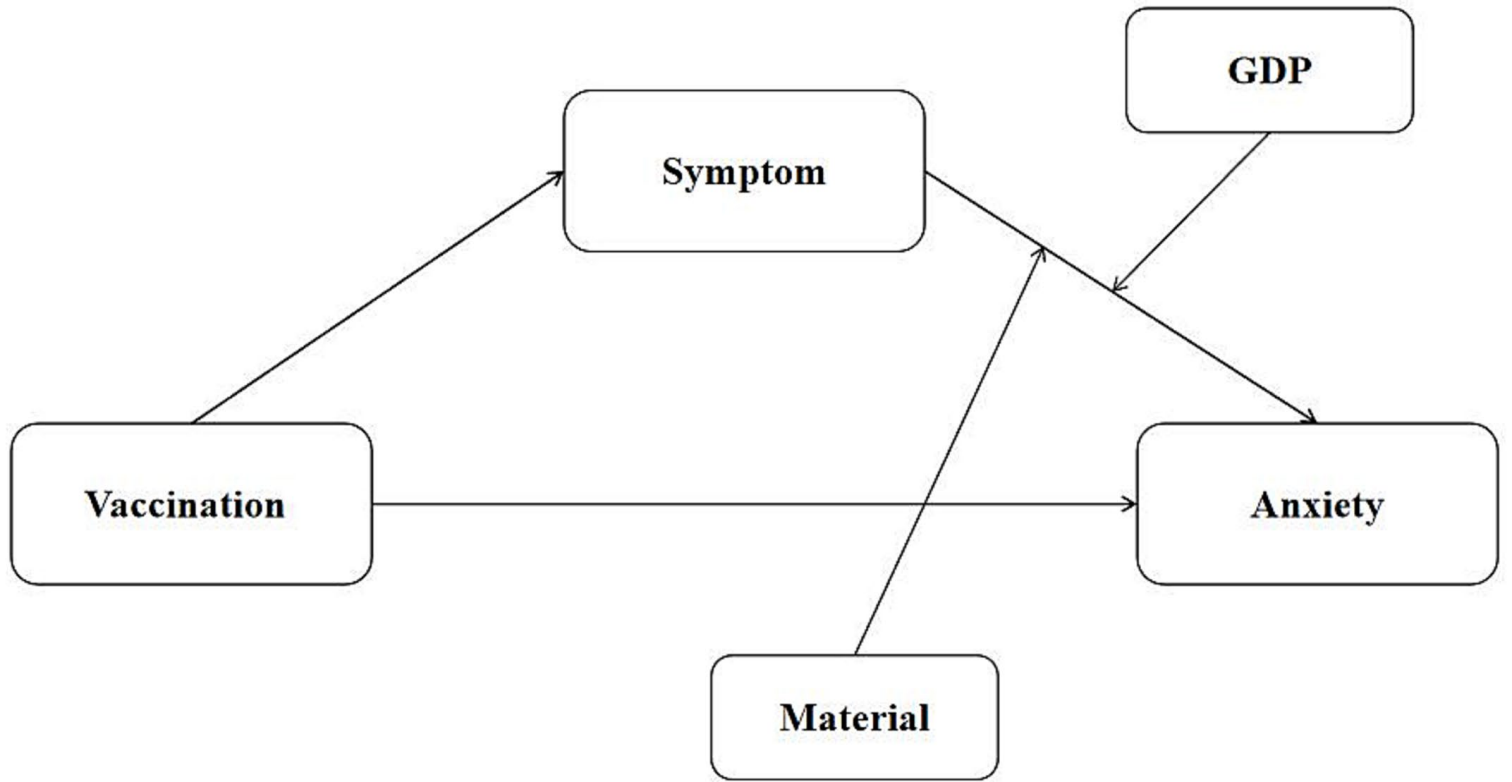
Conceptual moderated mediation model.

This study is theoretically grounded in the Conservation of Resources (COR) theory (17), which proposes that individuals seek to acquire and protect valuable resources such as health, material assets, and social support. Loss or threat of these resources induces stress and anxiety, whereas sufficient resources buffer against psychological distress. In this context, vaccination can be viewed as a protective health resource, while material reserves and socioeconomic conditions represent external resources that mitigate stress. In addition, the stress-buffering model (18) suggests that resource availability can weaken the impact of stressors (e.g., symptom severity) on mental health. Guided by these frameworks, this study constructed a moderated mediation model (Figure 1).

While extensive research has examined the impact of vaccination on anxiety, the psychological mechanisms—particularly in the context of multiple doses and evolving public health policies—remain inadequately elucidated. Existing studies often focus on isolated factors, lacking an integrated analysis of the complex interactions among vaccination doses, self-perceived symptoms, material reserves, and regional economic levels. This study constructed a moderated mediation model to investigate the relationship between vaccination and anxiety by examining the mediating role of the number of self-perceived symptoms and the moderating/mediating roles of material reserves and residential GDP. This model not only fills a critical gap in the understanding of the interplay between psychological mechanisms and structural factors but also provides a theoretical basis and practical implications for targeted public health interventions and resource allocation strategies.

## 2 Materials and methods

### 2.1 Participants

The data on emerging and sudden infectious diseases infections used in this study were collected through a web-based questionnaire from December 6, 2022, to January 14, 2023. A total of 42,912 valid questionnaires were collected. Study sample were excluded if they (1) were incomplete, (2) presented logical errors, and (3) were filled out by somebody other than the stated participant. Based on all these criteria, 40,302 questionnaires were finally included. The effective recovery rate of the questionnaire was 93.92% Informed consent was obtained from all participants.

### 2.2 Methods

The survey consisted of a self-designed questionnaire and a standardized psychometric scale assessing sociodemographic characteristics, prevalence of chronic disease, vaccination status, duration of emerging and sudden infectious diseases infection and symptoms, material reserves, and anxiety.

Sociodemographic characteristics included sex (Male/Female), age (20 ~ 29 years old, 30 ~ 39 years old, 40 ~ 49 years old, 50 ~ 59 years old, 60 ~ 69 years old, ≥70 years old) and residential area (urban/rural areas).

Participants were asked if they had any of the seven chronic diseases cited in the questionnaire (coronary heart disease, hypertension, diabetes, cerebrovascular disease, chronic obstructive pulmonary disease, malignant tumor, chronic kidney disease) or any other chronic disease, and if they had experienced any of the eight emerging and sudden infectious diseases symptoms included. Further, they were asked if they had a stock of relevant medications, disinfection products, personal protective equipment, or antigen tests (19).

The Generalized Anxiety Disorder 7 (GAD-7) is widely used to measure anxiety levels and assess the severity of generalized anxiety disorder, and was developed in 2006 by Spitzer, Kroenke, Williams, and colleagues at Columbia University. Participants’ degree of anxiety was measured using the GAD-7 scale, with total scores divided into five categories, indicating no (0 points), mild (1–4 points), moderate (5-9points), moderate to severe (10-14points), and severe (≥ 15 points) anxiety. Higher scores indicate a more severe anxiety condition (20).

### 2.3 Data analysis

Data were analyzed using R 4.2.3, with the *p*-value threshold for statistical significance set at 0.05 (two-tailed). Common method bias was assessed using Harman’s one-way test to determine the validity of the data. Multicollinearity was assessed through a multiple linear regression analysis with variance inflation factor (VIF); a VIF ≤ 5 was considered representative of non-collinearity (21). Descriptive analyses were then conducted, including for correlations between sociodemographic characteristics (sex, age, place of residence), number of vaccination doses, length of time since the last dose, number of self-perceived symptoms, level of material reserves, and anxiety. A chi-squared test was performed to determine whether anxiety scores varied according to demographic variables. Next, 95% CIs were assessed using Model 16 in PROCESS macro version 4.3 to examine moderated and moderated mediation effects with 10,000 bootstrap samples (22). A significant effect was observed when the CIs did not include 0. In addition, a simple slope analysis was used to explore the patterns of the significant moderating effects.

## 3 Results

### 3.1 Preliminary analyses

Descriptive statistics of participants’ sociodemographic characteristics and differences in anxiety levels, vaccination status, number of self-perceived symptoms, and level of material reserves during the emerging and sudden infectious diseases outbreak are presented in Table 1. A total of 14,268 (35.40%) participants were aged 50–59 years. Of these, 20,795 (51.60%) participants were female, and 28,013 (69.51%) participants resided in urban areas. Among the total sample 18,997 (47.14%) participants reported varying degrees of anxiety, and 31,466 (78.08%) had received booster shots. A total of 25,074 (62.21%) participants experienced self-perceived symptoms of emerging and sudden infectious diseases infection during the pandemic.

**Table 1:** Characteristics of the participants (*N* = 40,302).

Variable	Level	Anxiety					*χ*^2^ /P	Material					*χ*^2^ /P	Number of symptoms				*χ*^2^ /P
		No	Mild	Moderate	Moderately Severe	Severe		Nothing	Single	Double	Treble	All		Asymptomatic	1 ~ 4	5 ~ 9	≥10	
Gender	Female	10,098	5,815	1711	1732	1,439	329.57***	2,274	3,997	4,812	5,565	4,147	184.05***	6,841	2,643	6,164	5,048	691.26***
	Male	11,207	4,528	1,256	1,283	1,233		2,941	3,985	4,209	4,817	3,555		7,979	3,330	5,213	3,084	
Age (years)	20 ~ 29	843	502	115	101	127	139.46***	239	373	358	429	289	53.08***	330	172	357	322	519.4 ***
	30 ~ 39	3,029	1813	450	434	432		818	1,301	1,350	1,505	1,184		1993	889	1836	1,605	
	40 ~ 49	6,536	3,194	888	893	775		1,645	2,389	2,702	3,191	2,359		4,327	1794	3,595	2,534	
	50 ~ 59	7,662	3,540	1,078	1,085	903		1745	2,798	3,178	3,738	2,809		5,672	2066	3,910	2,551	
	60 ~ 69	2,646	1,076	368	408	357		632	939	1,170	1,250	864		2,299	702	1,273	772	
	≥70	589	218	68	94	78		136	182	263	269	197		607	214	270	212	
Region	Urban	14,348	7,406	2,161	2,180	1918	101.57***	2,867	4,778	6,289	7,698	6,381	1622.6***	9,638	3,922	8,164	5,930	404.19***
	Rural	6,957	2,937	806	835	754		2,348	3,204	2,732	2,684	1,321		5,590	1915	3,077	2066	
GDP	Higher	11,690	5,670	1,631	1,645	1,463	0.13	2,727	4,111	4,814	5,452	4,995	391.98***	8,193	3,138	6,278	4,405	13.41*
	Lower	9,615	4,673	1,336	1,370	1,209		2,488	3,871	4,207	4,930	2,707		7,035	2,699	4,963	3,591	
Dose of vaccine	0 dose	1,509	678	185	223	179	115.69***	355	594	637	675	513	71.36***	1,179	455	816	514	117.76***
	1 dose	265	159	55	60	56		75	115	135	141	129		282	159	256	191	
	2 dose	2,706	1,420	403	473	465		756	1,097	1,162	1,321	1,131		1868	785	1,574	1,288	
	3 dose	16,547	7,996	2,302	2,234	1930		3,986	6,093	7,014	8,118	5,798		11,558	4,319	8,436	5,860	
	4 dose	278	90	22	25	42		43	83	73	127	131		340	119	161	142	
Material	Nothing	2,339	1,181	405	530	760	859.45***											
	Single	4,168	2043	576	661	534												
	Double	4,677	2,354	752	687	551												
	Treble	5,722	2,758	740	690	472												
	All	4,399	2007	494	447	355												
Number of symptoms	Asymptomatic	9,056	3,493	942	919	818	3102.8 ***	2043	3,283	3,004	3,675	3,223	362.75***					
	1 ~ 4	3,810	1,198	325	281	223		728	1,353	1,345	1,442	969						
	5 ~ 9	5,603	3,125	974	903	636		1,293	1991	2,810	3,089	2058						
	≥10	2,339	2,319	951	1,151	1,236		1,280	1,375	1865	2040	1,436						

*p < 0.05, **p < 0.01, ***p < 0.001.

The correlations between the number of emerging and sudden infectious diseases vaccination doses, self-perceived symptoms, anxiety, household material reserves, and life satisfaction with residential GDP are shown in Table 2. Correlation analyses showed that the severity of anxiety in the last 2 weeks after full liberalization was negatively correlated with the number of vaccination doses (r = −0.03, *p* < 0.001) and greater material reserves (r = −0.09, *p* < 0.001), while positively correlated with the number of self-perceived symptoms after infection (r = 0.23, *p* < 0.001). Age, sex, and place of residence were added as covariates.

**Table 2:** Correlations of variables.

Variable	1	2	3	4	5	6	7	8
1. Vaccination	1.00							
2. Anxiety	−0.03***	1.00						
3. Symptoms	−0.02***	0.23***	1.00					
4. Material	0.01*	−0.09***	0.00	1.00				
5. Residential	0.04***	−0.05***	−0.11**	−0.20***	1.00			
6. Age	0.13***	−0.02***	−0.12***	0.01	0.01*	1.00		
7. Sex	−0.10***	0.08***	0.12***	0.05***	0.00	−0.11***	1.00	
8. GDP	0.07***	0.00	−0.02*	−0.07***	0.11***	−0.02***	0.00	1.00

*p < 0.05, **p < 0.01, ***p < 0.001.

### 3.2 The moderated mediation analysis

Table 3 shows the results of the moderated mediation analyses, where the independent variable was the number of emerging and sudden infectious diseases vaccination doses. Increasing the number of vaccination doses negatively predicted anxiety (β = −0.0249, 95%CI = [−0.0393, −0.0106]) and negatively predicted the number of self-perceived symptoms (β = −0.0367, 95%CI = [0.0228, 0.0506]). The number of self-perceived symptoms positively predicted recent anxiety (β = 0.2566, 95%CI = [0.2204, 0.2929]). When emerging and sudden infectious diseases vaccination was increased by one dose, associated anxiety decreased by 0.0249. The total effect of emerging and sudden infectious diseases vaccination doses on anxiety was significant (Effect = −0.0351, SE = 0.0076), as was the direct effect of the number of emerging and sudden infectious diseases vaccination doses (Effect = −0.0249, SE = 0.0073, 95%CI = [−0.0393, −0.0106]). The direct effect of the number of self-perceived symptoms on the indirect effect of the association between the number of emerging and sudden infectious diseases vaccination doses and anxiety was also significant (Effect = 0.0102, SE = 0.002, 95%CI = [0.0062, 0.0142]). This explained 29.1% of the variation in the total effect. These results support hypothesis H2.

**Table 3:** Results of the moderated mediation analysis.

Variable	Symptom				Anxiety			
	β	SE	95% CI		β	SE	95% CI	
			Lower	Upper			Lower	Upper
Sex	0.2754	0.0118	0.2523	0.2984	0.1169	0.0122	0.0929	0.1409
Age	−0.117	0.0056	−0.128	−0.106	0.03	0.0058	0.0186	0.0414
Region	−0.2678	0.0127	−0.2926	−0.243	−0.1028	0.0135	−0.1292	−0.0764
Vaccination	−0.0367	0.0071	0.0228	0.0506	−0.0249	0.0073	−0.0393	−0.0106
Symptom					0.2566	0.0185	0.2204	0.2929
Symptom × material					−0.035	0.0039	−0.0426	−0.0273
Symptom × GDP					0.033	0.0104	0.0127	0.0533
*R* ^2^	0.0384				0.0023			
*F*	386.434***				47.883***			

*p < 0.05, **p < 0.01, ***p < 0.001.

**Figure 2:**
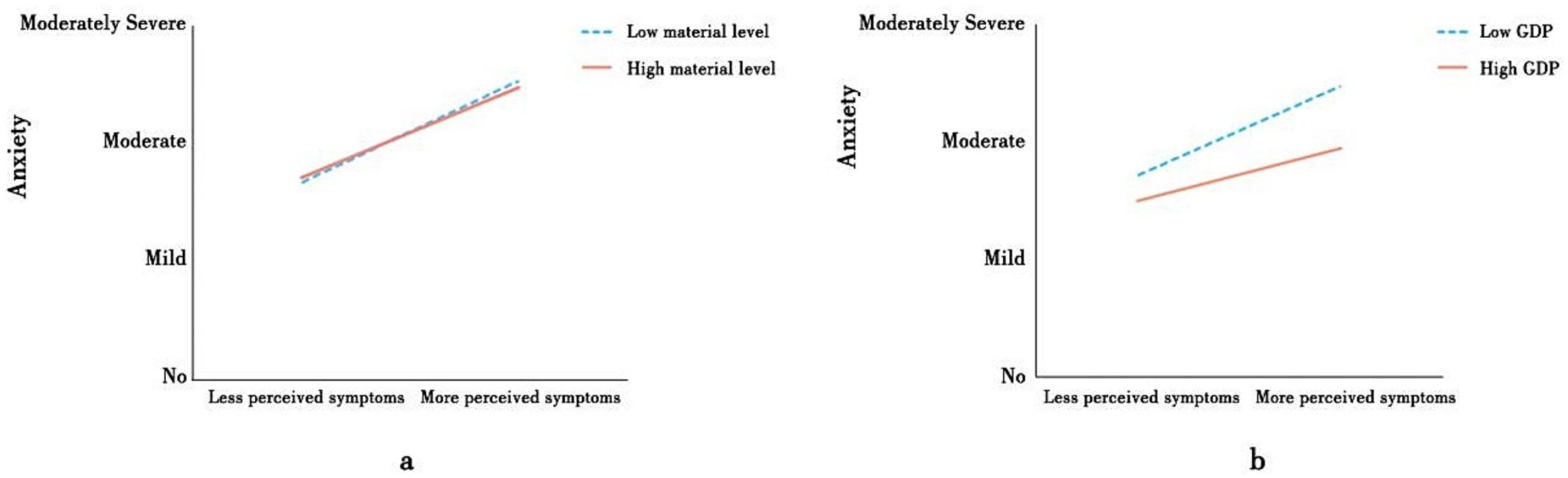
Moderating effect of affective forecasting. **(a)** Moderating effect of GDP. **(b)** Moderating effect of material.

A significant interaction was found between the number of self-perceived symptoms and personal material reserves (personal protection equipment, medication, sterilization products, antigen detection) and anxiety levels (β = −0.035, 95%CI = [0.0426, −0.0273]). Higher material reserves weakened the correlation between self-perceived symptoms and anxiety. A simple slope analysis was also conducted based on the number of types of relevant materials possessed by the participants (Figure 2a). As shown in Table 4, for participants with lower material reserves, more severe self-perceived symptoms were associated with more severe anxiety (Indirect Effect = 0.0115, 95%CI = [0.007, 0.016]). The proportion of the indirect effects relative to the number of self-perceived symptoms increased from 29.1 to 32.8%. However, this association was much weaker (Indirect Effect = 0.0076, 95%CI = [0.0046, 0.0107]) in participants with high material reserves (one SD above the mean). The proportion of indirect effects ranged from 29.1 to 21.7%. Thus, the correlation between the number of self-perceived symptoms and anxiety was stronger in the lower material reserves condition than in the high material reserves condition (β = −0.0013, 95%CI = [−0.0019, −0.0007]). These findings support H3.

**Table 4:** Indirect effect at specific levels of the moderator.

Moderating variables	Number of doses of vaccine → Number of symptoms → Anxiety			
	Effect	SE	95% CI	
			Lower	Upper
Low material level	0.0115	0.0023	0.007	0.016
High material level	0.0076	0.0015	0.0046	0.0107
Contrast	−0.0013	0.0003	−0.0019	−0.0007
Low GDP level	0.011	0.0022	0.0067	0.0154
High GDP level	0.0095	0.0019	0.0058	0.0133
Contrast	−0.0015	0.0005	−0.0006	−0.0026

A significant interaction was also found between the number of self-perceived symptoms and residential GDP with regard to anxiety after the implementation of the adjusted policy (β = 0.033, 95%CI = [0.0127, 0.0533]). Higher residential GDP weakened the correlation between the number of self-perceived symptoms and anxiety. A simple slope analysis was performed based on participants’ residential GDP (Figure 2b). Table 4 shows that, for participants in areas with lower residential GDP, a greater number of self-perceived symptoms was associated with more severe anxiety (Indirect Effect = 0.011, 95%CI = [0.0067, 0.0154]). The proportion of indirect effects relative to the number of self-perceived symptoms increased from 29.1 to 31.3%. However, this association was weaker for participants in areas with lower residential GDP (Indirect Effect = 0.0095, 95%CI = [0.0058, 0.0133]). The proportion of the indirect effects relative to the number of self-perceived symptoms decreased from 29.1 to 27.1%. Thus, the correlation between number of self-perceived symptoms and anxiety was stronger in areas with lower rather than higher residential GDP (β = −0.0015, 95%CI = [−0.0006, −0.0026]). These findings did not support H4.

## 4 Discussion

Since the World Health Organization declared emerging and sudden infectious diseases as a public health emergency of international concern at the end of January 2020 (23), China has implemented a strict prevention and control policy to manage the containment of the disease. The present study was conducted in December 2022, shortly after the declaration that these pandemic era controls would be lifted. Previous studies have shown a high prevalence of anxiety in patients with noninfectious chronic diseases. These concerns may be related to localized mobility constraints, inconvenient routine screening and medical care, as well as high economic costs. While a number of studies have explored the impact of emerging and sudden infectious diseases on mental health, few have focused on patients with chronic diseases or on the impact of emerging and sudden infectious diseases vaccinations on mental health (24, 25). A key contribution of this study is the examination of the association between vaccination and anxiety levels in chronically ill patients, and the exploration of self-perceived symptoms as a potential mediator within this relationship. This study systematically examined the relationship between the number of emerging and sudden infectious diseases vaccination doses and anxiety, as well as potential moderating and mediating mechanisms in a population of patients with chronic diseases following the introduction of the Class B infectious disease Class B Management policy. Our findings indicate that a higher number of vaccination doses was associated with lower levels of anxiety. Furthermore, this association was partially explained by self-perceived symptoms, and the relationship between these symptoms and anxiety was moderated by material reserves and residential GDP. The overall level of anxiety observed in this study was low, consistent with findings in studies conducted during the early Italian blockade, which also reported relatively low levels of mental health problems (26).

### 4.1 Vaccination and anxiety

Our results supported H1, indicating that a higher number of vaccination doses was associated with lower levels of anxiety. This finding aligns with population-wide strategies in many countries that promoted “booster” vaccinations to enhance immunity. For instance, a study from Italy found that moderate to severe anxiety was more common among individuals who had not received a COVID-19 vaccine (27) corroborating the inverse relationship we observed. It is plausible that as the number of vaccination doses increases, the level of individual immunity protection usually increases. Immunoprotection reduces the risk of infection, thereby providing a greater sense of security. Previous studies have shown that patients with chronic diseases are often more susceptible to serious infections because their immune systems are likely to be weaker. Multiple vaccination doses can provide additional protection and reduce the risk of serious illness and death. This finding has important implications for anxiety relief in chronically ill patients.

### 4.2 The mediating role of self-perceived symptoms

Self-perceived symptoms were used to explore the specific mechanisms through which vaccination affected anxiety during recurrent emerging and sudden infectious diseases outbreaks. Results showed that the number of self-perceived symptoms was significantly and positively correlated with anxiety levels. This finding is consistent with those of previous studies (28). More importantly, this study found that self-perceived symptoms mediated the relationship between vaccination and anxiety to some extent. In other words, the association between higher vaccination doses and lower anxiety was partly indirect, operating through a reduction in self-perceived symptoms. Self-perceived symptoms play an important bridging role, reflecting not only the relationship with vaccination doses, but also the relationship with anxiety, thus answering the question of “why” vaccination doses impact anxiety. Therefore, self-perceived symptoms are important endogenous factors influencing anxiety in patients with chronic diseases.

Increased vaccination status reduces symptoms following emerging and sudden infectious diseases infection. This finding is consistent with those of previous studies that have found a significant reduction in symptomatic infections after vaccination. Vaccination enables the immune system to recognize emerging and sudden infectious diseases antigens, allowing it to generate an immune response more quickly in the event of infection. This prevents the virus from replicating rapidly in the body, thereby reducing infection severity. For patients with chronic diseases, different self-perceived symptoms may increase anxiety because the patients may be unsure whether these symptoms are related to emerging and sudden infectious diseases or a health problem related to the underlying disease. This uncertainty may increase anxiety. Therefore, increasing the number of vaccination doses may help patients reduce their self-perceived symptoms and ultimately reduce anxiety.

In summary, for patients with chronic diseases, vaccination not only directly affects anxiety levels but also indirectly affects self-perceived symptoms. This mediating effect was found to be significant. This suggests that the effect of vaccination on anxiety is complex, and that anxiety levels can be reduced by increasing the number of vaccination doses (raising the level of immunization).

### 4.3 Moderating effects of material reserves and residential GDP

The findings of this study can be interpreted within the frameworks of the COR theory and the stress-buffering model. According to COR theory, individuals experience psychological distress when their valuable resources—such as health, material goods, and social support—are threatened or depleted. Adequate protective resources, including vaccination, sufficient material reserves, and higher socioeconomic conditions, can therefore reduce anxiety by enhancing perceived security and control. Consistent with the stress-buffering model, material reserves and residential GDP acted as buffers, mitigating the impact of self-perceived symptoms on anxiety. These results emphasize that both personal and environmental resources play crucial roles in protecting mental health during public health crises.

Our analysis revealed that the link between self-perceived symptoms and anxiety was stronger for individuals with limited material reserves. This finding is consistent with global reports of heightened anxiety during pandemics, particularly among vulnerable groups (29). As patients with chronic diseases are usually at greater health risk, they may be more worried about contracting emerging and sudden infectious diseases. Uncertainty increases when patients perceive multiple symptoms, and patients with poor material reserves may develop concerns about receiving the necessary assistance in a timely manner after the onset of symptoms.

Similarly, the association between self-perceived symptoms and anxiety was more pronounced for patients residing in areas with lower GDP. Regions with lower GDP often face a relative scarcity of medical resources, including hospital beds, equipment, and specialized personnel (30). People with chronic diseases may experience feelings of isolation and a lack of support systems, potentially exacerbating their anxiety. People with chronic disease in areas with lower residential GDP may also be more likely to face financial difficulties, including those related to healthcare costs and the risk of job loss. Anxiety levels may increase when individuals feel that their health is at risk in the face of significant financial burdens (31). The moderating effect of regional GDP on anxiety levels suggests that areas with lower economic resources may require additional support in terms of healthcare infrastructure and social services. This includes more healthcare facilities, trained personnel, and social support networks to reduce anxiety among patients with chronic diseases.

In summary, during the repeated Emerging and sudden infectious diseases outbreaks, vaccination influenced the anxiety of chronically ill patients through self-perceived symptoms; the second half of this process was moderated by material reserves and residential GDP. The community and social sectors concerned need to do a better job of testing the immunity levels of chronically ill patients; guaranteeing material reserves for high-risk groups during the winter and spring seasons, which are most conducive to the spread of emerging and sudden infectious diseases; and paying greater attention to the construction and development of healthcare to improve the psychological well-being of chronically ill patients. In essence, these findings advocate for a multi-faceted approach in public health policies, integrating vaccination campaigns and resource allocation strategies to safeguard the mental well-being of individuals facing the intersecting challenges of chronic disease and the emerging and sudden infectious diseases pandemic (or future health crises). These findings offer valuable insights for public health policymakers, healthcare providers, and community organizations.

### 4.4 Limitations

This study had some limitations. First, its cross-sectional design made it impossible to infer causal relationships between the variables; therefore, experimental and longitudinal designs should be utilized in future studies. Second, the self-reported questionnaire used in this study may have been influenced by social desirability, in addition to common methodological bias. Probability sampling techniques were not used to recruit subjects. However, because our survey was supported by the Shanxi Center for Disease Control and Prevention, the sample size was very large, which somewhat reduced the sample size imbalance caused by the use of non-probability sampling. Factors such as individual health literacy, prior exposure to health-related information, or cultural attitudes toward vaccination could have potentially impacted the results. Additionally, socio-economic status, access to healthcare resources, and the quality of healthcare infrastructure within residential areas may have introduced nuanced variations in the observed relationships. Future research endeavors could delve into these factors to provide a more nuanced understanding of the complex interplay between vaccination, anxiety, and associated elements among patients with chronic diseases. This exploration of alternative explanations will contribute to a more comprehensive and nuanced interpretation of the study’s findings, paving the way for refined interventions and policies.

## 5 Conclusion

This study highlights the need for further research on the mental health of patients with chronic diseases, particularly during health crises. Monitoring and adapting public health policies based on research findings can help improve the overall well-being of these vulnerable populations. Increasing the number of emerging and sudden infectious diseases vaccination doses has been shown to reduce the self-perceived symptoms of emerging and sudden infectious diseases infection, thereby mitigating anxiety during the disease outbreaks. Material reserves and residential GDP both moderate the relationship between vaccination and anxiety. These findings emphasize the importance of increasing vaccination rates to enhance population immunity levels when facing inevitable outbreaks of emerging and sudden infectious diseases infection following the adjustments made regarding disease prevention and control policies. It is important to maintain reserves at home to improve global mental health in the post-emerging and sudden infectious diseases era.

## Data Availability

The raw data supporting the conclusions of this article will be made available by the authors, without undue reservation.
